# Palindromic sequence impedes sequencing-by-ligation mechanism

**DOI:** 10.1186/1752-0509-6-S2-S10

**Published:** 2012-12-12

**Authors:** Yu-Feng Huang, Sheng-Chung Chen, Yih-Shien Chiang, Tzu-Han Chen, Kuo-Ping Chiu

**Affiliations:** 1 Genomics Research Center, Academia Sinica, 128 Academia Rd., Sec. 2, Nankang, Taipei 115, Taiwan; 2 NGS core, TechCom division, National Taiwan University, Taiwan; 3 Institute of Zoology, National Taiwan University, Taiwan

## Abstract

**Background:**

Current next-generation sequencing (NGS) platforms adopt two types of sequencing mechanisms: by synthesis or by ligation. The former is employed by 454 and Solexa systems, while the latter by SOLiD system. Although the *pros *and *cons *for each sequencing mechanism have more or less been discussed in a number of occasions, the potential obstacle imposed by palindromic sequences has not yet been addressed.

**Methods:**

To test the effect of the palindromic region on sequencing efficacy, we clonally amplified a paired-end ditag sequence composed of a 24-bp palindromic sequence flanked by a pair of tags from the *E. coli *genome. We used the near homogeneous fragments produced from *Mme*I digestion of the amplified clone to generate a sequencing library for SOLiD 5500xl sequencer.

**Results:**

Results showed that, traditional ABI sequencers, which adopt sequencing-by-synthesis mechanism, were able to read through the palindromic region. However, SOLiD 5500xl was unable to do so. Instead, the palindromic region was read as miscellaneous random sequences. Moreover, readable tag sequence turned obscure ~2 bp prior to the palindromic region.

**Conclusions:**

Taken together, we demonstrate that SOLiD machines, which employ sequencing-by-ligation mechanism, are unable to read through the palindromic region. On the other hand, sequencing-by-synthesis sequencers had no difficulty in doing so.

## Background

Next-Generation Sequencing (NGS) promises to provide robust sequencing platforms for basic biological researches as well as clinical and molecular diagnoses [1-4]. Currently, the sequencing market is dominated by 3 NGS lineages: Roche's 454, Illumina's Solexa, and Life Technologies' SOLiD. In general, the cost for 454 is much higher than the other two, but its sequence length can easily reach 400 bps, followed by the Solexa lineage and then SOLiD, which can only produce short reads up to 75 bp.

The sequencing chemistries of these three systems more or less differ. 454 uses emulsion PCR to amplify temples on beads followed by pyrosequencing with DNA polymerase [5]. Solexa lineage adopts bridge PCR to amplify temples on slide followed by colorimetric sequencing with DNA polymerase. As such, both 454 and Solexa lineages adopt "sequencing-by-synthesis" approach. On the other hand, although SOLiD systems also use emulsion PCR to amplify templates on beads, its sequencing is extended by two unique steps: first by using oligos to hybridize to the target in the single-stranded template, followed by using ligase to seal the nick between the 5' of the growing strand and the 3' of the oligo. This sequencing approach, which grows in 3' to 5' direction, is coined as "sequencing-by-ligation" mechanism. The most evident difference between sequencing-by-synthesis and sequencing-by-ligation is that the former uses DNA polymerase to incorporate complementary nucleotides to the elongating strand, while the latter uses ligase to seal the junction between the elongating strand and the newly incorporated complementary oligonucleotides. Since DNA polymerase is an essential enzyme in the cell, the former is considered as a more natural approach compared with the latter.

Potential underrepresentation of GC-rich regions have been reviewed for both sequencing-by-synthesis and sequencing-by-ligation mechanisms [6]. However, the potential obstacles imposed by palindromic regions have never been addressed. A palindromic sequence is a nucleic acid sequence that reads the same no matter from the 5' end of the sequence itself or from the 5' end of its complementary strand. It has a potential to form a hairpin structure [7]. The success of ligation has to rely on the availability of its complementary strand. Given the fact that short stretches of palindromic sequences may form hairpin structures in the single-stranded template, palindromic sequences which naturally exist in genomes or in RNA sequences (esp. mRNAs or miRNAs) may become regional obstacles for sequencing-by-ligation sequencers, causing problems in genome assembly and introducing bias in transcriptome analysis. Thus, it is an important issue to further understand the potential obstacles caused by palindromic sequences, especially for the sequencing-by-ligation mechanism.

In this article, we used centralized palindromic sequence to study the effects of a palindromic sequence on sequencing. We constructed a single clone containing a palindromic region flanked by two tag sequences (~18 bp), amplified the clone, digested with *Mme*I, and constructed a sequencing library from the *Mme*I fragments. The library was sequenced by SOLiD 5500xl sequencer. Here we report the results, indicating the incapability for a sequencing-by-ligation machine to read through the palindromic region.

## Methods

### Clone preparation and sequencing library construction for the palindromic library

Paired-end ditag DNA fragment of 5'-GGCAA TGGCA CCATC GCT*AG TCGGA GTCTG CGCAG ACTCC GACT*C CACGA CCGCT GAGGT T-3' was cloned into yT&A vector (Cat. YC013, Yeastern Biotech. Co., Ltd.) and verified by traditional Sanger sequencing using ABI PRISM^® ^96-capillary 3730xl DNA Analyzer (Notice that this step proved the capability of a traditional sequencing-by-synthesis method to sequence through the palindromic region). This clone contains a centralized palindromic sequence (26 bp, 5'-AGTCG GAGTC TGCGC AGACT CCGAC T-3') flanked by two tags originated from the *E. coli *genome: Tag1 (18 bp, 5'-GGCAA TGGCA CCATC GCT-3') and Tag2 (17 bp, 5'-CCACG ACCGC TGAGG TT-3', or 5'-AACCT CAGCG GTCGT GG-3' on the complementary strand which was sequenced by SOLiD 5500xl sequencer), making up a total length of 61 bp. The inserted DNA in yT&A vector was amplified with PCR using M13 forward (5'-GTTTT CCCAG TCACG AC-3') and reverse (5'-TCACA CAGGA AACAG CTATG AC-3') primers and EconoTaq™ PLUS GREEN 2X Master Mix (Lucigen Corp.). PCR amplified fragments were digested with *Mme*I, end-repaired, A-tailed and ligated with P1 and P2 sequencing adaptors to make the palindromic library for sequencing by SOLiD 5500xl. The quality and quantity of library were checked by Agilent 2100 Bioanalyzer and Roche LightCycler^® ^480 II Real-Time PCR system according to the manufacturer's instructions.

### Sequencing by SOLiD 5500xl

The fragment library prepared above was sequenced by SOLiD 5500xl for 7 ligation cycles (X 5 primers) to obtain 35 bp sequences. By using Exact Call Chemistry (ECC) module, we directly obtained nucleotide sequences, instead of colour space information.

### Bioinformatics analysis

Raw sequences were processed for quality control [8]. Briefly, sequences with N, polyA, polyT, polyG, polyC, and PCR primer sequence were removed. Various quality values (QVs) were used, depending on what types of sequences we wanted to observe. Only the qualified sequences were used for analysis.

**Figure 1:**
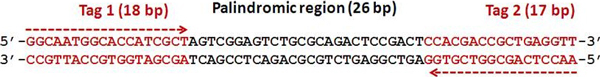
**The original construct**. The construct contains a 26-bp palindromic sequence in the middle flanked by two tags originated from *E. coli *genome.

Tag sequences located at the 5' terminals were supposed to be followed by their corresponding palindromic sequences. To accommodate potential sequencing error and the effect of palindromic sequence, we aligned the leading 18 bp of each read against the tag sequences and obtained two major groups, starting with either Tag1 or Tag2 (see Figure 1). The procedure included: 1) sort the leading 18 bp; 2) count each unique reads; 3) align unique reads against the Tag1 and Tag2; and 4) count the number of reads with exact or partial tag sequences. This stepwise procedure was executed in sequential order to ensure the accuracy of sequence alignment.

## Results and discussion

### Library construction

The original clone was a double-stranded construct containing a centralized palindromic sequence (26 bp) flanked by two tags, Tag1 (18 bp) and Tag2 (17 bp), originated from the *E. coli *genome, making up a total length of 61 bp (Figure 1). For testing the capability of a SOLiD machine to read through the palindromic region, a sequence length of 35 bp is long enough for our purpose, and there was no need to sequence the full construct.

Notice that the internal adaptor region is sealed by two *Mme*I sites (TCCGAC) each followed by an extra T previously used for stick-end ligation to *E. coli *genomic fragments. This extra T can be assigned to the tag region, but it was not originated from the *E. coli *genome.

**Figure 2:**
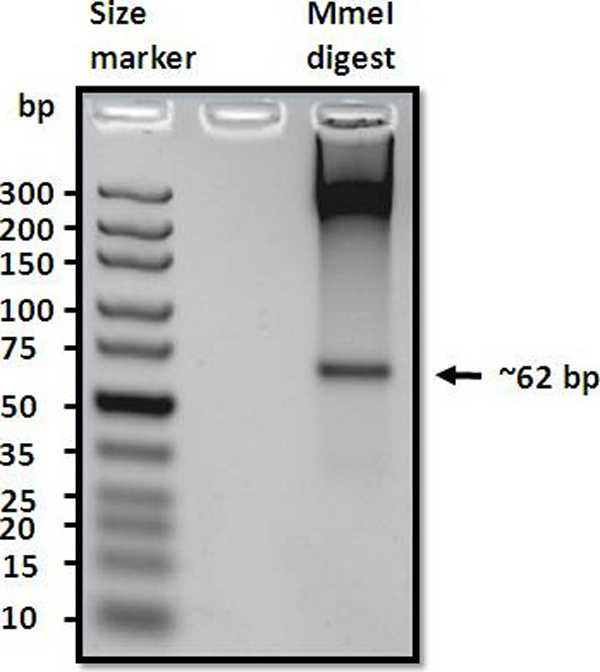
**Agarose gel electrophoresis of *Mme*I digest**. *Mme*I digest of *E. coli *amplified yT&A vector was subjected to 4% agarose gel electrophoresis. The arrow indicates the location of the *Mme*I fragment.

Due to the imprecision of *Mme*I digestion, incubation of *Mme*I with PCR amplified fragments produced a near homogeneous population of *Mme*I fragments with a few bp variations at either end (Figure 2). However, the length variation normally does not exceed +2 bp [9], resulting in a distinguishable ~62 bp band in agarose gel. *Mme*I fragments excised from agarose gel was purified and used for sequencing library construction.

### Sequencing results

As mentioned above, there was no need to sequence the full construct (total of ~62 bp) as this study was set out to test the capability of SOLiD 5500xl to read through a palindromic structure. As such, we run 7 cycles for each one of the five primers to make up a total of 35 bp sequence length. The 35 bp sequences produced by SOLiD 5500xl were expected to contain the 5' end of either tag followed a ~17 bp of the palindromic region.

The statistic numbers associated to sequence data processing are shown in Table 1. Among the qualified reads, 40.05% matched Tag1 and 31.37% matched Tag2. Notice that tag sequences with extra bases at the 5' end were excluded, causing a potential underestimation of the percentages. However, we were focusing on the readability of the palindromic region. This minor issue can be ignored.

**Table 1:** Library statistics

Reads	Read count	
Raw reads	116,094,828	

Qualified reads	110,694,180	

		Percentage (compared with qualified reads)

Tag1-containing reads	44,334,569	40.05%

Tag2-containing reads	34,723,418	31.37%

Total of Tag1/Tag2-containing reads	79,057,987	71.42%

**Figure 3:**
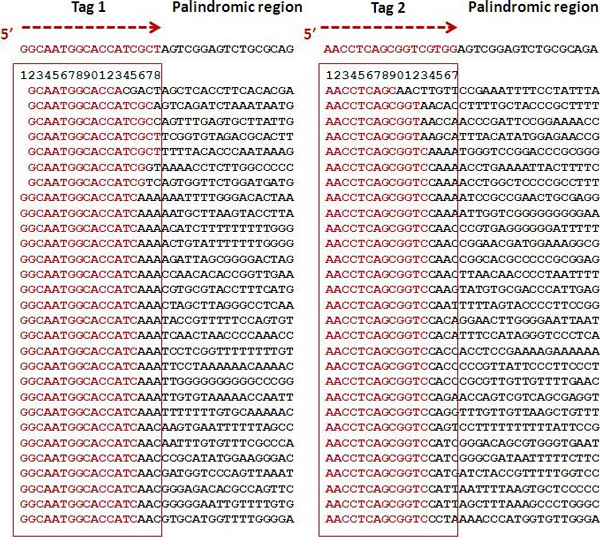
**Quality sequence reads sorted on tag sequences**. Sequences produced from SOLiD 5500xl were processed and sorted based on Tag 1 and Tag 2 sequences shown on top. Complete tag sequences are enclosed within red boxes, which are followed by the palindromic region. Bases matching to the expected tag or palindromic sequences are highlighted in red, while the unmatched bases are highlighted in black.

Sequence sorting and alignment revealed a clear pattern: a region of tag sequences followed by a region of random sequences failing to match the palindromic sequence (Figure 3). As such, SOLiD 5500xl failed to read through the palindromic region.

**Figure 4:**
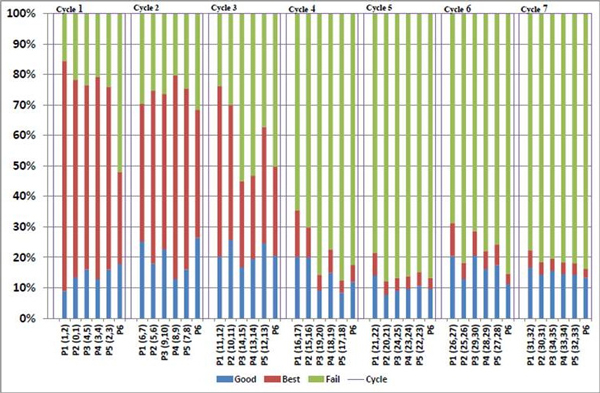
**The decline of the good-and-best beads ratio observed during the sequencing of the palindromic library by SOLiD 5500xl**. The beads quality is indicated by colour: blue for good beads, red for best beads and green for bad beads. Their percentages are shown in Y-axis. Only sequences from the good beads (blue bar) and best beads (red bar) can be used for analysis. A total of five primers (P1-5) have to be used for each sequencing run. (Notice that P6 is an ECC primer, which was not used for sequence reading.) In this study, each sequencing run lasted for 7 ligation cycles to complete the 35 bp sequence length. The positions of each two-base encoding for each primer cycle are shown within the parenthesis right behind the primer. In the library, every template comprises a tag of 17-18 bp, followed by the palindromic region of 26 bp, and then another tag (also 17-18 bp). During the first two cycles, corresponding to the tag region, the percentages of good and best beads seemed to be quite normal. The initial decline of good-and-best beads ratio was seen in P3(14, 15) and P4(13,14), and an overwhelming decline observed across all primers started from the fourth cycle when sequencing entered the palindromic region.

**Figure 5:**
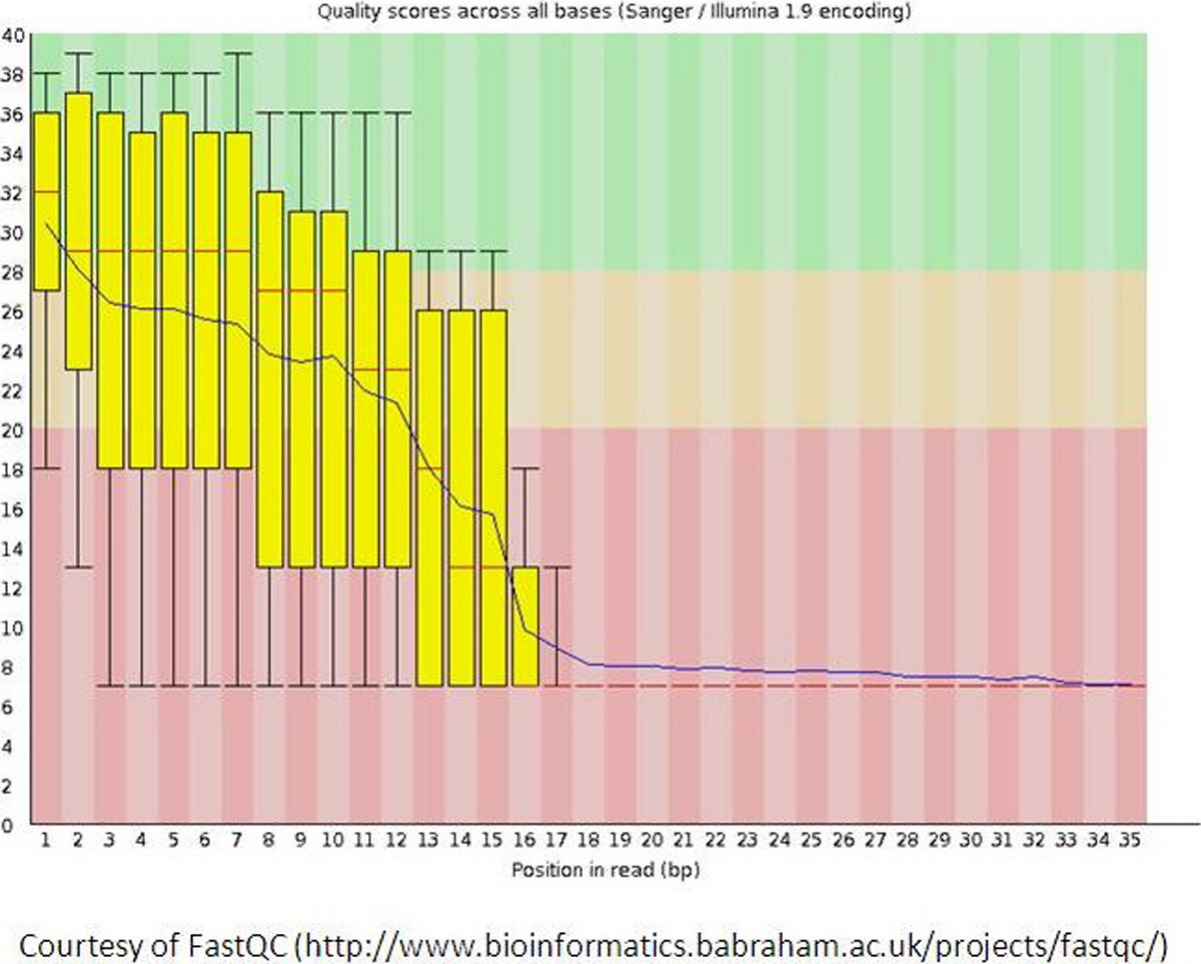
**The decline in quality value observed in the palindromic library when sequenced by SOLiD 5500xl**. This figure was generated by using FastQC package available online. Quality values are shown in Y-axis, while the base positions are shown in X-axis. The QV baseline was found to be 7, as retrieved directly from the sequencer.

The incapability of SOLiD 5500xl to read through the palindromic region was further indicated by two pieces of evidence: drop in good-and-best beads ratio (Figure 4) and drop in QV (quality value) (Figure 5).

### A dramatic decline in good-and-best beads ratio was observed in the third cycle

Positions read by sequencing-by-ligation follows the unique two-base encoding pattern. As defined by the design of the oligo probes, each 2-base reading is followed by a 3-base leap (gap). To compose a complete reading, each sequencing run utilizes 5 primers (P1-5) and there is a single-base shifting between primers (Figure 4): Primer 1 reads positions (1,2) ... (6,7) ... and so on; Primer 2 reads positions (0,1)...(5,6)... and so on; Primer 3 reads positions (4,5)...(9,10)... and so on; Primer 4 reads positions (3,4)...(8,9)... and so on; Primer 5 reads positions (2,3)...(7,8)... and so on. P6, an ECC primer not for actual sequence reading, is not included.

SOLiD systems use good-and-best beads ratio as an indicator to evaluate the quality of the library. High good-and-best beads ratio implies high library quality. During the sequencing of the palindromic library, we found that the ratios of good-and-best beads for the first 2 cycles seemed quite normal, but started to decline for P3 and P4 during their third cycle, followed by a global decline during the fourth cycle (Figure 4). This result provided further evidence showing the incapability of SOLiD 5500xl sequencer for reading through the palindromic region in the library.

### A gradual decline in quality value completely quenched sequence quality to the manufacturer-defined ground level at ~17th position

As indicated previously, the palindromic region starts from position 18 or 19. We noticed a gradual decline of quality value, and it completely dropped to the manufacturer-defined baseline QV 7, at position 17 - about a couple of bases before the palindromic region (Figure 5). As such, the palindromic region would disappear if the QV cutoff was set to be 8 or above. That is, the faulty/random palindromic sequences can only be seen with QV cutoff equal to or below 7.

### Local double-stranded hairpin structure was supposed to result in the inaccessibility of the single-stranded template, leading to the failure in sequencing

**Figure 6:**
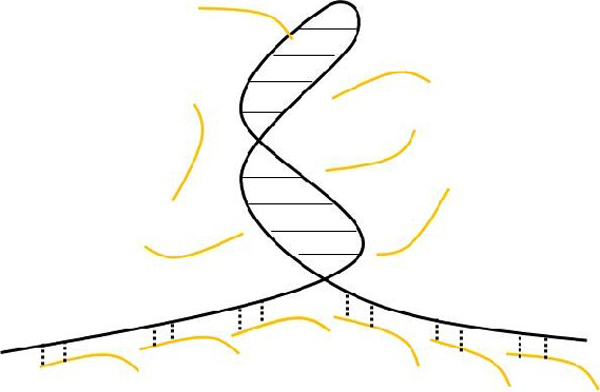
**Schematic drawing showing the inaccessibility of template in the palindromic region**. Local hairpin may form in the palindromic region, making the single-stranded template become inaccessible. Black solid line, DNA template; orange lines, oligos; black dashed lines, successful hybridization between DNA template and oligos.

For sequencing-by-ligation mechanism, templates need to be made in single-stranded form for oligos to hybridize. As such, single-stranded templates were generated right before every sequencing run. However, the presence of a palindromic sequence in the template is very likely to induce the formation of a double-stranded hairpin the in the single-stranded context, making the template become inaccessible (Figure 6). We believe this is the major reason causing the interruption of sequencing in the palindromic region. Schematic drawing showing the inaccessibility of template in the palindromic region. Local hairpin may form in the palindromic region, making the single-stranded template become inaccessible. Black solid line, DNA template; orange lines, oligos; black dashed lines, successful hybridization between DNA template and oligos.

## Conclusions

The original construct was also sequenced by ABI PRISM^® ^96-capillary 3730xl DNA Analyzer, which adopts sequencing-by-synthesis approach, indicating that palindromic sequences imposed no difficulty for the sequencing-by-synthesis sequencers. However, as indicated by our results, the SOLiD 5500xl sequencing-by-ligation sequencer was unable to read through the palindromic region presumably due to the plausible formation of hairpin structure in the palindromic region.

As such, sequencing-by-ligation-based sequencers may not be suitable for genome assembly, because palindromes of various sizes are known to present in both prokaryotic and eukaryotic genomes. On the other hand, this type of sequencers may remain suitable for sequencing transcriptomes and small DNA molecules of which hairpin structures are less likely to be present. Moreover, the length of a palindrome is expected to be negatively correlated with its readability, although not in proportional.

## Competing interests

The authors declare that they have no competing interests.

## Authors' contributions

YFH analyzed sequence data, generated some figures, and wrote part of the manuscript. SCC prepared sequencing library, generated a few figures, and wrote part of the manuscript. YSC analyzed some sequence data. THC was in charge of sequencing. KPC directed the study, guided data analysis, and wrote the manuscript.
